# Integrating quantitative traits and *growth hormone* gene polymorphism in Indonesian crossbred chickens for genetic improvement and marker-assisted selection

**DOI:** 10.14202/vetworld.2025.2169-2180

**Published:** 2025-08-02

**Authors:** Depison Depison, Gushairiyanto Gushairiyanto, Ratna Sholatia Harahap, Raden Abdul Muthalib, Abdul Azis, Yun Alwi, Sarwo Edy Wibowo

**Affiliations:** 1 Department of Animal Husbandry, Faculty of Animal Science, Universitas Jambi, Jambi, 36361, Indonesia; 2 Department of Animal Health, Faculty of Animal Science, Universitas Jambi, Jambi, 36361, Indonesia

**Keywords:** chicken, crossbreed, egg characteristic, genetic selection, *growth hormone* gene, growth performance, morphometry

## Abstract

**Background and Aim:**

Local Indonesian chickens possess valuable dual-purpose traits for both meat and egg production, but exhibit lower productivity compared to commercial breeds. Genetic enhancement through selective crossbreeding and molecular marker analysis, such as *growth hormone* (*GH*) gene polymorphism, offers a strategy to improve performance traits. This study aimed to characterize quantitative traits and analyze *GH* gene polymorphism in crossbred chickens resulting from mating Arab chickens with five indigenous breeds.

**Materials and Methods:**

Five local breeds, Kampung Super, Sentul, Bangkok, Kampung, and Merawang, were each crossed with Arabian chickens using a 1:7 male-to-female ratio. Phenotypic evaluations included body weight (BW), weight gain, and 18 morphometric traits measured at various ages. Egg traits were monitored for 4 weeks. *GH* gene polymorphism was identified in 500 crossbred individuals using polymerase chain reaction-restriction fragment length polymorphism (PCR-RFLP) with the AluI enzyme restriction. Data were analyzed through a one-way analysis of variance and General Linear Models to determine phenotypic and genotypic associations.

**Results:**

Significant differences (p < 0.05) in BW, BW gain, morphometric size, and egg traits were observed among parental and crossbred groups. Kampung Super × Arab chickens showed the highest performance across growth and reproductive metrics. The highest weight gain occurred between 2 and 3 months of age. Back height emerged as a key morphometric indicator of growth differences. PCR-RFLP revealed *GH* gene polymorphism with three genotypes: (+/+), (+/−), and (−/−). The (+/+) genotype had a significant (p < 0.05) positive impact on BW, weight gain, and back height. All populations were in Hardy–Weinberg equilibrium, and polymorphic information content values (~0.437) indicated moderate genetic diversity.

**Conclusion:**

This study is the first comprehensive integration of morphometric, phenotypic, and GH genotypic data in Indonesian crossbred chickens. The findings support the implementation of marker-assisted selection to enhance growth traits in breeding programs. Future work should assess multi-generational effects and integrate additional molecular markers to optimize breeding strategies across tropical poultry systems.

## INTRODUCTION

Indigenous chickens serve as a valuable genetic resource and hold significant potential for the deve-lopment of superior poultry lines. However, systematic breeding programs aimed at enhancing these lines remain largely undeveloped. As a result, their genetic potential, particularly for traits related to produc-tivity, adaptability, and disease resistance, remains underutilized [1]. Most local chicken breeds are dual-purpose, supplying both meat and eggs [2]. Among them, Arabian chickens are known for their relatively high egg production (~250–260 eggs/year) compared to other free-range breeds [3]. Despite this advantage, Arabian chickens display inferior body weight (BW), growth rate, and morphometric dimensions when compared to Kampung Super, Sentul, and Bangkok breeds [4–10]. Crossbreeding Arabian chickens with local Indonesian lines, such as Kampung Super, Sentul, Bangkok, Kampung, and Merawang, presents a strategic approach to combine desirable traits, including high egg production and improved growth performance. The success of these crosses can be evaluated thr-ough offspring performance indicators, including BW, weight gain, and body conformation. Growth-related traits, being quantitative and heritable, are effective markers for early selection [8]. Understanding the genetic basis of these traits is crucial for sustainable improvement programs [5,9,11,12]. Key growth per-formance indicators include BW, weight gain, and various morphometric measurements [13]. The ado-ption of molecular-assisted selection can expedite the identification of superior genotypes at early deve-lopmental stages. The *growth hormone* (*GH*) gene plays a pivotal role in regulating growth, metabolism, and reproductive functions. It significantly impacts egg production, carcass yield, and skeletal development in chickens. Specifically, the chicken *GH* gene influences key physiological and production traits such as egg number [14], muscle and carcass development [2], reproductive performance in Mazandaran chickens [15], growth rate in Thai broilers [16], skeletal developm-ent [17], and postnatal growth in broiler lines such as Hubbard F15 and Cobb E [18]. Despite the recognized potential of indigenous chickens for improving genetic diversity and productivity in tropical poultry systems, systematic efforts to enhance their economic traits through targeted crossbreeding and molecular appr-oaches remain limited in Indonesia. While some local breeds such as Kampung Super, Sentul, and Bangkok exhibit superior growth traits, and Arabian chi-ckens are notable for their high egg-laying capacity, comprehensive evaluations that integrate phenotypic performance with genotypic data, particularly the *GH* gene are scarce. Moreover, few studies have examined the direct association between *GH* gene polymorphisms and growth-related phenotypes in crossbred chickens derived from local Indonesian lines. This represents a significant knowledge gap in the development of efficient marker-assisted selection (MAS) strategies for indigenous poultry improvement.

This study aimed to evaluate growth perfor-mance, morphometric traits, and egg characteristics in several Indonesian crossbred chickens resulting from crosses between Arabian chickens and five local breeds: Kampung Super, Sentul, Bangkok, Kampung, and Merawang. Furthermore, it sought to identify *GH* gene polymorphisms using polymerase chain reaction-restriction fragment length polymorphism (PCR-RFLP) and assess their association with key growth traits in crossbred populations. The findings are intended to support future breeding programs by providing foun-dational data for the implementation of MAS and genetic enhancement of native chicken lines in tropical regions.

## MATERIALS AND METHODS

### Ethical approval

All animal handling procedures were approved by the Ethical Clearance Committee of the Faculty of Animal Science, Jambi University, Indonesia (Approval No. 01/01/UN21.7/ECC/2024).

### Study period and location

The study was conducted from March 2023 to June 2024 at the Department of Animal Husbandry, Faculty of Animal Science, University of Jambi, Muaro Jambi, Indonesia.

### Animals and breeding management

**Figure 1:**
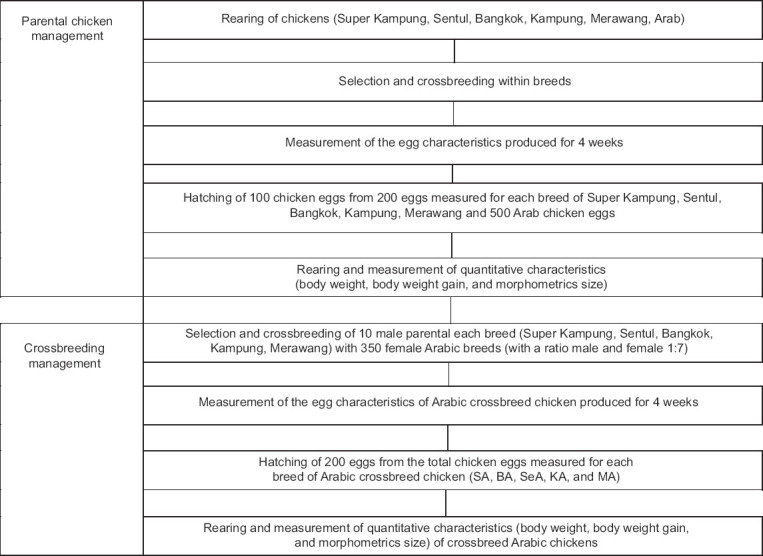
The flowchart of the rearing and breeding management of parental and crossbred chickens.

**Figure 2:**
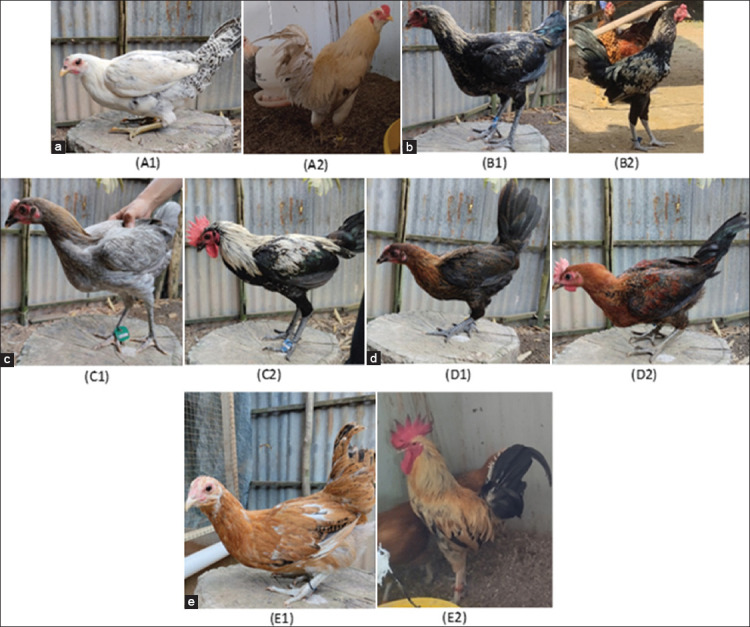
Phenotype characteristics of Indonesian crossbreed Arabic chicken (a) Kampung Super Arab, (b) Bangkok Arab, (c) Sentul Arab, (d) Kampung Arab, and (e) Merawang Arab; 1. Female, 2. Male.

The experiment involved six chicken breeds: Super Kampung, Sentul, Bangkok, Kampung, Merawang, and Arabian. A total of 100 eggs from each local breed and 500 eggs from Arabian chickens were incubated for hatching. From these, ten males from each local breed and 350 Arabian females were selected based on quantitative traits for crossbreeding. The mating ratio was maintained at one male to seven females to produce five hybrid groups: Super × Arab (SA), Bangkok × Arab (BA), Sentul × Arab (SeA), Kampung × Arab (KA), and Merawang × Arab (MA) (Figure 1). The phenotype of crossbred chickens is presented in Figure 2. All birds were fed a standard commercial broiler diet and provided ad libitum access to clean water. Housing conditions were maintained uniformly to minimize environmental effects and ensure a reliable genotype-phenotype association. For molecular analysis, blood samples were randomly collected from 100 crossbred chickens through wing vein puncture.

### Measurement of egg characteristics and growth performance

Quantitative traits were evaluated in both parent and crossbred chickens. Egg characteristics, including egg weight, length, width, and circumference were recorded daily over a 4-week laying period. Growth performance was assessed by measuring BW at 4-week intervals from hatch to 16 weeks (BW0, BW4, BW8, BW12, BW16), with each measurement replicated 3 times. BW gain was calculated for four intervals: 0–4 weeks, 4–8 weeks, 8–12 weeks, and 12–16 weeks. Morphometric traits, including beak length, head circumference, chest width, and tibia length, were measured at 16 weeks using a digital caliper.

### *GH* gene polymorphism detection through PCR-RFLP

Genomic DNA was extracted from blood sam-ples using the Wizard Genomic DNA purification kit (Promega, USA) according to the manufacturer’s instructions. PCR amplification of the *GH* gene was performed using specific primer sets (Table 1) on an Esco PCR thermocycler (Aeris PCR Thermal Cycler, Esco Micro Pte. Ltd., Singapore). The 15 μL PCR mixture contained 0.2 μL each of forward and reverse primers, 7.5 μL of MyTaq HS RedMix (Bioline, UK), and 6.1 μL of nuclease-free water. Amplification conditions inclu-ded an initial denaturation at 95°C for 5 min, followed by 35 cycles of 95°C for 45 s, 57°C–60°C for 45 s, and 72°C for 45 s, with a final extension at 72°C for 5 min. PCR products were visualized on 1.5% agarose gels stained with FluoroSafe (1^st^ BASE Biochemicals Product, Malaysia) and electrophoresed using Mupid-exU at 100 V for 35 min. The amplified fragments were digested with the AluI restriction enzyme at 37°C for 4 h and visualized on 2.5% agarose gels under ultraviolet transillumination. Genotypes were identified by comparing DNA fragment patterns against a 100-bp marker.

**Table 1:** Length and location of the *growth hormone* (*GH*) gene and primers used for PCR.

Segment position	Product length (bp)	Type of primer	Sequen (5’- 3’)	Annealing temperature (°C)
17–929	912	GH1 Fwd.	AAGCACTGCCTGTGAAGCTC	60.7
		GH1 Rev.	CGGGCCATTCTATGAGTCTG	60.6
961–1571	611	GH2 Fwd.	TCAACCCCTTGCACTTGTC	59.7
		GH2 Rev.	GTTGGCAAACAGGTTGGAGA	61.1
1217–1837	621	GH3 Fwd.	CTGAGCTGTTCCCAGTCCTC	60.0
		GH3 Rev.	CAACCCGTGTCTTTCTGCAT	59.6
2117–2633	517	GH4 Fwd.	CCAGGCTGCGTTTTGTTACT	60.3
		GH4 Rev.	TCCCTTCTTCCAGGTCCTTT	60.0
3186–3915	730	GH5 Fwd.	GGGCAGTTTGAGCTGGTG	60.4
		GH5 Rev.	ATGGTGCAGTTGCTCTCTCC	60.4

Fwd=Forward, Rev=Reverse, PCR=Polymerase chain reaction

### Statistical analysis

Growth performance data were analyzed using a one-way analysis of variance, followed by Duncan’s multiple range test for *post hoc* comparisons (p < 0.05). The statistical model used was:

Yij = μ + Bi + eij

Where Yij is the observed trait (e.g., BW and egg trait), μ is the overall mean, Bi is the fixed effect of breed, and eij is the residual error. The association between GH genotypes and growth traits was assessed using general linear models. Genotypic and allelic frequencies were calculated using the Nei and Kumar [19] method. The Hardy–Weinberg equilibrium was tested using the Chi-square (χ^2^) test, as described by Hartl and Clark [20]. Genetic diversity indicators, including observed heterozygosity, expected heterozygosity, and polymorphic information content (PIC), were calculated to assess the informativeness of the markers. This study provides a rare phenotypic–genotypic correlation analysis in Indonesian poultry, contributing to future MAS frameworks.

## RESULTS AND DISCUSSION

### Egg characteristics of parental and crossbred chickens

The analysis revealed significant differences (p < 0.05) in egg characteristics among all parental chicken breeds (Table 2). Kampung Super chickens had the highest egg traits, while Arabian chickens exhibited the lowest. Among the crossbred groups, SA chickens exhibited the most favorable egg traits, while MA chickens had the least favorable. These variations are likely attributable to the male parental lines used in the crosses. Strain and male line use significantly influence BW and morphometric size [21], and the accuracy of selection in males tends to be greater than in females [22].

**Table 2:** Average characteristics of parental eggs and the results of crosses.

Egg characteristics	Breeds						p-value

	Parental						

	Kampung super (n = 200)	Bangkok (n = 200)	Sentul (n = 200)	Kampung (n = 200)	Merawang (n = 200)	Arab (n = 500)	
Egg weight (g)	59.33 ± 0.74^a^	55.43 ± 1.21^ab^	53.99 ± 2.42^b^	45.71 ± 3.16^c^	43.22 ± 1.51^c^	42.03 ± 1.13^d^	0.022
Egg length (mm)	60.73 ± 1.84^a^	56.73 ± 0.28^ab^	54.67 ± 1.21^b^	51.09 ± 1.05^c^	46.11 ± 0.65^d^	44.03 ± 1.13^d^	0.016
Egg circumference (mm)	155.40 ± 1.40^a^	148.77 ± 1.11^ab^	145.32 ± 0.58^b^	132.51 ± 1.27^c^	113.54 ± 1.31^d^	103.03 ± 1.21^e^	0.001
Egg width (mm)	49.49 ± 1.72^a^	47.38 ± 1.31^ab^	46.28 ± 0.61^ab^	42.08 ± 0.09^b^	36.16 ± 1.14^c^	33.03 ± 0.21^d^	0.018

**Egg characteristics**	**Crossbred result**						**p-value**

	**SA (n = 355)**	**BA (n = 404)**	**SeA (n = 357)**	**KA (n = 394)**	**MA (n = 319)**		

Egg weight (g)	49.76 ± 0.62^a^	48.43 ± 1.01^ab^	47.31 ± 2.12^b^	44.25 ± 1.29^c^	42.68 ± 1.16^d^		0.032
Egg length (mm)	53.21 ± 0.13^a^	52.82 ± 0.89^ab^	51.33 ± 0.22^ab^	49.23 ± 0.25^b^	45.31 ± 0.65^c^		0.001
Egg circumference (mm)	135.78 ± 0.83^a^	129.39 ± 0.13^b^	124.75 ± 0.21^bc^	119.01 ± 0.32^c^	108.68 ± 0.41^d^		0.025
Egg width (mm)	43.24 ± 1.13^a^	41.21 ± 1.14^ab^	39.72 ± 1.14^ab^	37.89 ± 0.19^b^	34.69 ± 1.14^c^		0.006

SA=Kampung Super × Arab native chicken, BA=Bangkok × Arab, SeA=Sentul × Arab, KA=Kampung × Arab, MA=Merawang × Arab. Superscripts a, ab, b, c, d, and e=Significantly different at 5% (p < 0.05) for the egg characteristics trait between each chicken breeds; The numbers shown in parentheses are the number of individuals with the specified breed

The egg traits recorded in this study exceeded those reported in earlier studies. Previously docum-ented average egg weights include 41.12 g for Kam-pung Super [23], 46.57 g for Bangkok [24], 42.7 g for Kampung [25], and 42.93 g for Merawang chickens [10]. Sentul chickens were reported to have average egg weights of 49.62 g and 50.12 g [5,7]. For Kampung chickens, mean values were as follows: egg weight, 44.15 ± 1.44 g; egg length, 50.68 ± 1.85 mm; width, 40.22 ± 0.65 mm; and circumference, 126.28 ± 2.15 mm [9].

Variations in egg characteristics are primarily driven by genetic background, rearing practices, and environmental conditions. Factors such as maternal age, stage of laying, and nutrition management can influence egg weight [26, 27]. Genetically, differences in egg size are linked to variation in albumen and yolk content, with larger eggs generally containing greater proportions of both components [28].

### BW of parental and crossbred chickens

Significant differences (p < 0.05) in BWs were observed across all age intervals among both parental and crossbred chickens (Table 3). While parental chickens were generally heavier than the crossbreds, the crossbred groups still showed an improved BW compared to pure Arabian chickens. This improve-ment suggests a heterotic effect resulting from crossbreeding. Noor [29] defines heterosis as enhanced performance in crossbreds compared to their parent lines.

**Table 3:** Average body weight of parents and their crosses.

Age	Breeds						p-value

	Parental weight (g)						

	Kampung super (n = 89)	Bangkok (n = 87)	Sentul (n = 76)	Kampung (n = 84)	Merawang (n = 85)	Arab (n = 453)	
DOC	45.91 ± 1.46^a^	42.15 ± 2.05^ab^	38.62 ± 1.95^b^	36.25 ± 0.72^b^	34.01 ± 1.45^c^	33.63 ± 0.45^c^	0.025
1 Months	484.08 ± 50.20^a^	456.36 ± 52.03^ab^	431.63 ± 34.62^b^	389.53 ± 42.70^bc^	333.15 ± 23.44^c^	270.63 ± 10.23^d^	0.013
2 Months	878.03 ± 82.03^a^	847.03 ± 71.72^a^	789.28 ± 21.09^b^	721.27 ± 60.56^c^	651.28 ± 71.69^c^	592.23 ± 32.45^d^	0.001
3 Months	1376.31 ± 60.56^a^	1308.31 ± 82.14^a^	1230.36 ± 77.44^ab^	1134.45 ± 72.28^b^	1046.01 ± 59.96^c^	882.53 ± 12.45^c^	0.034
4 Months	1744.71 ± 296.17^a^	1614.68 ± 197.60^a^	1374.37 ± 164.97^b^	1303.32 ± 253.91^b^	1107.70 ± 161.44^c^	923.34 ± 126.08^d^	0.008

**Age**	**Crossbred weight (g)**						**p-value**

	**SA (n = 194)**	**BA (n = 185)**	**SeA (n = 188)**	**KA (n = 173)**	**MA (n = 177)**		

DOC	39.93 ± 2.01^a^	37.68 ± 1.97^b^	35.82 ± 1.87^c^	34.76 ± 1.62^c^	32.89 ± 2.12^d^		0.001
1 Months	378.79 ± 22.10^a^	365.43 ± 28.43^ab^	355.06 ± 23.62^b^	332.25 ± 27.70^c^	319.255 ± 24.11^d^		0.003
2 Months	757.41 ± 36.04^a^	733.01 ± 30.73^b^	703.23 ± 31.19^c^	652.33 ± 33.56^d^	625.16 ± 34.98^e^		0.008
3 Months	1211.61 ± 36.94^a^	1157.11 ± 30.79^b^	1101.45 ± 32.89^c^	1060.75 ± 39.86^d^	1014.49 ± 35.98^e^		0.007
4 Months	1427.60 ± 179.38^a^	1372.59 ± 110.14^b^	1247.43 ± 139.20^c^	1201.91 ± 163.43^d^	1154.11 ± 94.42^e^		0.007

SA=Kampung Super × Arab native chicken, BA=Bangkok × Arab, SeA=Sentul × Arab, KA=Kampung × Arab, MA=Merawang × Arab. Superscripts a, ab, b, c, d, and e=Significantly different at 5% (p < 0.05) for the body weight trait between each chicken breeds; the numbers shown in parentheses are the number of individuals with the specified breed, DOC=Day-old chick

The day-old chick (DOC) weights for BA, SeA, KA, and MA chickens in this study were higher than those reported in several prior studies. For example, Rowiyanti *et al*. [30] found average DOC weights of 35.22 g, 151.52 g, and 729.39 g at 1, 2, and 3 months, respectively, in Bangkok × Kampung crosses. Subiharta and Prabowo [31] reported 28.9 ± 1.4 g for DOCs in Sentul–Kampung Unggul Balitnak crosses. Kampung Super chickens weighed 40.03 g at DOC, 349.47 g at 1 month, and 837.98 g at 2 months [4], while Rahayu *et al*. [6] found slightly higher values.

Bangkok chickens reached 1244.81 g at 3 months, Sentul chickens 1281.49 g, Kampung chickens 1134.51 g [9] and 1261.01 g at 4 months [8], and Merawang chickens 1193.75 g at 4 months [10]. These variations are likely due to differences in breed composition and rearing environment. Growth traits in chickens are influenced by additive, dominance, and epistatic gene interactions [32, 33].

Mancinelli *et al*. [34] predicted growth perfor-mance using the Gompertz model and highlighted heterosis in crossbreeds. Hailemariam *et al*. [35] also showed that additive, maternal, and heterotic effects influence growth and production traits. Environmental factors, such as lighting, ambient temperature, and transportation handling, further modulate BW [36].

### BW gain from parental and crossbred chickens

The mean difference test revealed that BW gain across all breeds differed significantly (p < 0.05) at each age interval for both parental and crossbred chickens (Table 4). Peak weight gain occurred between 2 and 3 months, followed by the 1–2 month and DOC–1 month intervals. Growth typically decelerates between 3 and 4 months, coinciding with the onset of sexual maturity. This decline continues between 3 and 5 months as mat-urity progresses [9].

**Table 4:** Average body weight gain of parents and their crosses.

Age	Breeds						p-value

	Parental body weight gain (g)						

	Kampung super (n = 89)	Bangkok (n = 87)	Sentul (n = 76)	Kampung (n = 84)	Merawang (n = 85)	Arab (n = 453)	
DOC-1 Months	398.17 ± 11.23^a^	385.21 ± 16.67^a^	366.01 ± 14.77^b^	324.28 ± 12.72^c^	281.14 ± 18.07^d^	273.21 ± 20.54^e^	0.018
1–2 Months	434.95 ± 11.22^a^	420.67 ± 22.17^a^	385.65 ± 23.18^b^	361.74 ± 14.21^bc^	338.13 ± 20.54^c^	321.13 ± 23.54^d^	0.003
2–3 Months	497.28 ± 50.92^a^	461.28 ± 45.94^b^	441.08 ± 41.83^c^	413.18 ± 47.41^d^	394.73 ± 57.24^d^	360.73 ± 51.24^e^	0.005
3–4 Months	306.71 ± 25.04^a^	278.37 ± 36.75^b^	238.06 ± 25.46^b^	192.68 ± 22.61^c^	164.26 ± 26.11^d^	148.80 ± 22.11^d^	0.010

**Age**	**Crossbred body weight gain (g)**						**p-value**

	**SA (n = 194)**	**BA (n = 185)**	**SeA (n = 188)**	**KA (n = 173)**	**MA (n = 177)**		

DOC-1 Months	338.86 ± 12.43^a^	327.75 ± 14.29^ab^	319.24 ± 14.77^b^	297.49 ± 12.72^c^	242.66 ± 18.07^d^		0.004
1–2 Months	378.62 ± 21.22^a^	367.58 ± 22.17^ab^	348.17 ± 13.18^bc^	320.08 ± 14.21^c^	314.91 ± 20.54^d^		0.008
2–3 Months	434.20 ± 30.92^a^	424.10 ± 25.94^ab^	398.22 ± 20.18^bc^	408.42 ± 27.01^c^	389.33 ± 22.24^c^		0.001
3–4 Months	235.99 ± 24.04^a^	215.48 ± 28.75^a^	145.98 ± 23.46^b^	141.16 ± 23.61^b^	139.62 ± 25.11^b^		0.002

SA=Kampung Super × Arab native chicken, BA=Bangkok × Arab, SeA=Sentul × Arab, KA=Kampung × Arab, MA=Merawang × Arab. Superscripts a, ab, b, bc, c, d, and e=significantly different at 5% (p < 0.05) for the body weight gain trait between each chicken breed. The numbers in parentheses represent the number of individuals with the specified breed, DOC=Day-old chick

Kampung Super chickens showed BW gains of 202.00 g (DOC–1 month), 280.3 g (1–2 months), and 416.1 g (2–3 months) [37], and Rahayu *et al*. [6] reported 334.21 g, 415.57 g, and 456.88 g in the same intervals. Sentul chickens had average gains of 364.65, 383.11, 497.75, and 219.73 g from DOC to 4 months [5], and Irmaya *et al*. [8] noted gains of 184.31, 414.72, 388.57, and 165.92 g.

Kampung chickens gained 335.43, 359.26, and 387.39 g between DOC and 3 months [7], similar to Utama *et al*. [38], who found gains of 329.61, 355.07, and 374.55 g. Irmaya *et al*. [8] observed 282.6 g gain between 3 and 4 months. Merawang chickens had gains of 173.90, 351.48, 391.03, and 154.94 g over the same time frame [10].

These differences are influenced by genetic diversity and environmental variables. Depison and Gushariyanto [7] noted that both genetic background and husbandry practices affect BW gain. In addition, Mariandayani *et al*. [39] emphasized the role of insulin-like growth factor 2 in regulating growth performance.

### Morphometric sizes of parental and crossbred chickens

Table 5 indicates that morphometric traits varied significantly (p < 0.05) among parental breeds. Kam-pung Super chickens exhibited the largest morphometric size, followed by Bangkok, Sentul, Kampung, Merawang, and Arabian chickens. Among the crossbreds, SA and BA chickens had significantly greater dimensions than SeA, KA, and MA, which may be due to their comparatively heavier BWs. As reported by Prawira *et al*. [9], larger BW correlates with larger morphometric measurements.

**Table 5:** Average morphometric sizes of parental and crossbreed chickens.

Morphometric size (mm)	Parental					

	Super	Bangkok	Sentul	Kampung	Merawang	Arab
BeL	52.09 ± 1.21^a^	46.49 ± 2.14^b^	41.14 ± 1.27^b^	36.90 ± 2.54^c^	31.25 ± 2.74^d^	29.07 ± 1.63^d^
BW	13.96 ± 1.01^a^	12.26 ± 1.13^a^	11.06 ± 1.83^b^	10.98 ± 3.51^b^	9.96 ± 1.66^c^	7.37 ± 0.54^d^
HL	60.5 ± 0.71^a^	56.67 ± 0.57^b^	51.71 ± 0.43^c^	48.01 ± 0.85^c^	42.93 ± 0.76^d^	35.18 ± 1.67^e^
HH	45.38 ± 2.64^a^	42.85 ± 2.66^a^	39.39 ± 1.44^b^	35.82 ± 2.35^c^	31.77 ± 1.65^d^	28.81 ± 1.56^d^
HC	128.51 ± 5.40^a^	123.42 ± 5.5^b^	121.24 ± 2.17^b^	117.32 ± 7.62^c^	109.89 ± 5.61^d^	100.97 ± 4.23^e^
NL	161.29 ± 7.39^a^	156.45 ± 7.74^b^	152.82 ± 5.79^c^	147.02 ± 7.47^c^	136.23 ± 7.28^d^	99.78 ± 5.80^e^
NC	107.59 ± 5.23^a^	102.39 ± 6.35^a^	95.90 ± 5.09^b^	89.81 ± 5.79^c^	83.12 ± 5.64^d^	73.56 ± 4.03^e^
WL	226.70 ± 6.62^a^	220.1 ± 6.16^b^	218.16 ± 7.19^b^	196.04 ± 7.53^c^	189.86 ± 7.41^d^	174.69 ± 6.47^e^
BL	295.94 ± 7.23^a^	289.83 ± 8.63^a^	278.06 ± 7.08^b^	265.03 ± 8.12^c^	256.72 ± 6.34^d^	219.52 ± 5.12^e^
BH	335.79 ± 7.76^a^	331.38 ± 6.23^a^	317.47 ± 7.45^b^	305.63 ± 7.43^c^	286.16 ± 6.71^d^	249.42 ± 5.12^e^
CL	145.20 ± 8.38^a^	140.32 ± 8.55^b^	137.92 ± 6.57^c^	134.11 ± 8.38^d^	128.33 ± 6.35^e^	106.42 ± 4.84^e^
CW	77.16 ± 5.11^a^	71.03 ± 6.47^b^	60.24 ± 4.27^c^	54.81 ± 3.51^d^	49.91 ± 3.27^e^	42.58 ± 4.50^e^
SL	83.42 ± 4.04^a^	78.31 ± 3.71^b^	72.95 ± 4.48^c^	67.97 ± 3.67^d^	59.83 ± 4.21^e^	52.32 ± 4.17^e^
SC	58.13 ± 2.01^a^	52.97 ± 2.5^b^	48.09 ± 3.79^b^	44.84 ± 3.35^c^	41.9 ± 3.28^c^	35.67 ± 1.84^d^
TL	148.22 ± 2.34^a^	143.23 ± 3.74^a^	135.30 ± 3.09^b^	130.43 ± 3.92^b^	123.13 ± 6.44^c^	107.47 ± 5.49^d^
TC	129.23 ± 3.14^a^	127.21 ± 3.83^a^	121.63 ± 3.07^b^	116.08 ± 3.48^b^	84.42 ± 5.25^c^	78.42 ± 5.25^d^
LFT	76.28 ± 5.04^a^	71.91 ± 5.15^a^	68.66 ± 6.07^b^	61.42 ± 6.36^b^	55.10 ± 5.43^c^	51.89 ± 3.45^d^
PBD	16.01 ± 0.66^a^	15.12 ± 0.53^a^	14.23 ± 0.47^a^	13.84 ± 0.67^a^	13.51 ± 0.39^a^	13.12 ± 0.39^a^

**Body size (mm)**	**Crossbreed**					

	**SA (n = 65)**	**BA (n = 73)**	**SeA (n = 78)**	**KA (n = 65)**	**MA (n = 69)**	

BeL	36.40 ± 1.81^a^	36.22 ± 1.81^a^	34.27 ± 1.84^b^	34.18 ± 1.60^b^	33.81 ± 1.80 ^b^	
BW	8.70 ± 0.45^a^	8.55 ± 0.65^a^	7.91 ± 0.48^b^	8.00 ± 0.38^b^	7.84 ± 0.49 ^b^	
HL	42.45 ± 2.01^a^	41.32 ± 1.96^a^	40.68 ± 1.81^b^	40.87 ± 1.8^b^	39.75 ± 1.82^b^	
HH	35.18 ± 1.85^a^	34.56 ± 1.57^a^	33.23 ± 1.57^b^	33.37 ± 1.63^b^	32.88 ± 1.81^b^	
HC	121.57 ± 4.93^a^	121.31 ± 4.36^a^	116.00 ± 5.31^b^	116.02 ± 5.16^b^	115.97 ± 4.73^b^	
NL	128.42 ± 9.23^a^	128.54 ± 12.71^a^	124.10 ± 5.45^b^	123.32 ± 11.04^b^	123.22 ± 6.71^b^	
NC	93.27 ± 9.98^a^	92.32 ± 7.69^a^	86.88 ± 9.95^b^	87.30 ± 8.28^b^	86.39 ± 7.47^b^	
WL	206.39 ± 21.35^a^	205.79 ± 20.09^a^	198.06 ± 23.83^b^	198.49 ± 22.20^b^	197.06 ± 21.05^b^	
BL	229.18 ± 14.01^a^	228.87 ± 17.42^a^	221.41 ± 13.37^b^	218.71 ± 14.25^c^	215.78 ± 13.24^d^	
BH	268.88 ± 13.43^a^	269.52 ± 24.66^a^	254.03 ± 14.54^b^	252.10 ± 15.76^c^	251.02 ± 12.17^d^	
CL	123.58 ± 7.51^a^	122.03 ± 7.35^a^	114.58 ± 12.30^b^	112.71 ± 7.29^c^	111.14 ± 7.46^d^	
CW	62.35 ± 4.73^a^	61.56 ± 4.46^a^	57.50 ± 4.45^b^	55.07 ± 4.70^c^	53.42 ± 4.05^d^	
SL	84.16 ± 7.25^a^	84.04 ± 7.09^a^	81.73 ± 6.62^b^	79.84 ± 6.40^c^	78.53 ± 6.80^d^	
SC	51.98 ± 2.27^a^	51.01 ± 2.47^a^	46.68 ± 3.82^b^	45.36 ± 2.70^c^	43.91 ± 3.00^d^	
TL	126.97 ± 7.31^a^	125.91 ± 7.22^a^	120.56 ± 6.34^b^	118.63 ± 7.23^c^	116.08 ± 6.48^d^	
TC	111.72 ± 7.18^a^	111.62 ± 8.92^a^	106.35 ± 8.23^b^	106.26 ± 7.08^b^	106.01 ± 6.88^b^	
LFT	63.75 ± 4.91^a^	63.08 ± 4.47^a^	60.88 ± 5.13^b^	60.13 ± 4.60^b^	59.01 ± 3.76^b^	
PBD	17.56 ± 0.57^a^	17.84 ± 1.26^a^	16.40 ± 0.71^b^	16.24 ± 1.01^b^	16.17 ± 0.56^b^	

SA=Super × Arab native chicken, SeA=Sentul × Arab, BA=Bangkok × Arab, KA=Kampung × Arab, MA=Merawang × Arab. The vector of average values from the two groups of local Indonesian chickens observed included the following: BeL=Beak length, BW=Beak width, HL=Head length, HC=Head circumference, HH=Head height, NL=Neck length, NC=Neck circumference, WL=Wing length, BL=Body length, BH=Back height, CL=Chest length, CW=Chest width, SL=Shank length, SC=Shank circumference, TL=Tibia length, TC=Tibia circumference, LFT=Longest finger length, PBD=Pubic bone distance. Different superscript letters on the same row for each strain mean significantly different (p < 0.05)

These findings align with Rahayu *et al*. [6], who observed larger morphometric sizes in Super chic-kens compared to Bangkok chickens. Kampung Super chickens had larger sizes than both Kampung and Arabian breeds, while Sentul chickens were taller than Arabian and Merawang breeds [4].

Genetic inheritance largely determines morph-ometric variation. Differences in strain have been shown to influence body dimensions in chickens [38], including laying hens [40]. Since all chickens in this study were raised under uniform conditions, the observed morphometric differences are likely gene-tically driven, corroborating conclusions of earlier studies by Putri and Depison [4] and Milas *et al*. [41]. Nonetheless, morphometric traits are also affected by environmental influences such as housing location and management practices [42].

### Principal component analysis (PCA) of morphometric size in crossbreeds

PCA was applied to determine the major cont-ributors to morphometric variation. The first principal component explained most of the variation, 92.20% in SA, 95.50% in BA, 85.70% in SeA, 92.40% in KA, and 83.20% in MA chickens, primarily reflecting size. The trait with the highest eigenvector in all groups was back height (Tpu). The second principal component, which represented shape, contributed smaller pro-portions: 3.20%, 1.30%, 4.40%, 1.20%, and 1.80%, respectively (Table 6).

**Table 6:** Similarities in morphometric size and shape after crossing several chicken lines with Arabian chickens.

Crossbreed	Similarity			KT (%)	Λ
SA	Size	=	0.215BeL + 0.227BW + 0.234HL + 0.239HH + 0.2387HC + 0.235NL + 0.232NC−0.233WL + 0.242BL + 0.**243BH** + 0.235CL + 0.240CW + 0.233SL + 0.238LS + 0.239TL + 0.238TC + 0.239LFT−0.242PBD	92.20	16.56
	Shape	=	−0.531BeL + 0.092BW + 0.065HL + 0.009HH + 0.030HC−0.205NL−0.415NC + **0.312W**L−0.116BL + 0.042BH−0.345CL + 0.183CW + 0.220SL + 0.108LS−0.158TL + 0.207TC + 0.221LFT−0.220PBD	1.80	0.319
BA	Size	=	0.233BeL + 0.231BW + 0.232HL + 0.238HH + 0.237HC + 0.233NL + 0.232NC + 0.231WL + 0.239BL + **0.240BH** + 0.231CL + 0.239CW + O.239SL + 0.238SC + 0.239TL + 0.239TC + 0.238LFT + 0.235PBD	95.50	17.195
	Shape	=	−0.285BeL−0.192BW + 0.286HL−0.056HH + 0.123HC−0.359NL + 0.085NC−0.411WL + 0.114BL−0.118BH + **0.522CL** + 0.063CW−0.180SL + 0.282SC−0.009TL + 0.207TC + 0.050LFT−0.126PBD	1.30	0.227
SeA	Size	=	0.205BeL + 0.208BW + 0.226HL + 0.233HH + 0.234HC + 0.234NL + 0.236NC + 0.225WL + 0.248BL + **0.252BH** + 0.226CL + 0.251CW + 0.235SL + 0.235SC + 0.250TL + 0.251TC + 0.250LFT + 0.250PBD	85.70	15.422
	Shape	=	−0.493BeL−0.025BW−0.189HL−0.154HH−0.116HC + 0.130NL + 0.053NC + 0.351WL−0.173BL + 0.035BH + **0.443CL**−0.040CW + 0.035SL + 0.500SC−0.106TL + 0.063TC−0.166LFT−0.129PBD	4.40	0.784
KA	Size	=	0.235BeL + 0.223BW + 0.236HL + 0.236HH + 0.239HC + 0.233NL + 0.238NC + 0.235WL + 0.240BL + **0.243BH** + 0.236CL + 0.232CW + 0.236SL + 0.237SC + 0.238TL + O.234TC + 0.235LFT + 0.236PBD	92.40	16.635
	Shape	=	−0.124BeL−0.522BW + 0.137HL + 0.249HH + 0.071HC−0.328NL + 0.074NC−0.358WL−0.149BL−0.101BH + **0.251CL** + 0.311CW + 0.098SL−0.108SC + 0.142TL + 0.286TC + 0.209LFT−0.162PBD	1.20	0.222
MA	Size	=	0.201BeL + 0.228BW + 0.235HL + 0.235HH + 0.237HC + 0.241NL + 0.239NC + 0.231WL + 0.243BL + **0.256BH** + 0.248CL + 0.239CW + 0.240SL + 0.239SC + 0.241TL + 0.239TC + 0.240LFT + 0.205PBD	83.20	14.975
	Shape	=	−0.618BeL−0.047BW−0.296HL−0.156HH−0.055HC + 0.104NL + 0.053NC + **0.424WL**−0.021BL + 0.320BH−0.154CL−0.039SL + 0.003SC−0.087PTL + 0.076TC + 0.064LFT + 0.402PBD	3.20	0.575

SA=Super × Arab native chicken, SeA=Sentul × Arab, BA=Bangkok × Arab, KA=Kampung × Arab, MA=Merawang × Arab, Bold value=The highest value of each

parameter indicates that the parameter is a characteristic for each size and shape, BeL=Beak Length, BW=Beak Width, HL=Head Length, HC=Head Circumference, HH=Head Height, NL=Neck Length, NC=Neck Circumference, WL=Wing Length, BL=Body Length, BH=Back Height (BH), CL=Chest Length, CW=Chest Width, SL=Shank Length, SC=Shank Circumference, TL=Tibia Length, TC=Tibia Circumference, LFT=Longest Finger Length, and PBD=Pubic Bone Distance

For SA and MA chickens, wing length (Psa) cont-ributed the most to body shape, while in BA, SeA, and KA chickens, chest length (PD) was the primary contributor. The distribution pattern of eigenvalues across components (ƛ1 > ƛ2 > ƛ3…ƛn > 0) followed a typical decreasing trend.

These results differ from those of Irmaya *et al*. [8], who reported chest length as the main size component and shank length as the primary shape trait across several Indonesian chicken lines. This variation highlights the breed-specific nature of mor-phological contributions to overall conformation.

### Polymorphisms and associations of *GH* genes with growth performance

PCR-RFLP analysis confirmed polymorphism in the *GH* gene across all crossbred groups (SA, BA, SeA, KA, MA), with three genotypes identified: (+/+), (+/−), and (−/−) (Table 7). According to Allendorf *et al*. [43] and Nei and Kumar [19], a gene is considered polymorphic when no single allele frequency exceeds 0.99. The Chi-square test confirmed that *GH* gene distributions in all populations adhered to Hardy–Weinberg equilibrium, indicating random mating. As stated by Klug [44], genetic equilibrium is maintained when mating is unrestricted and external genetic influences, such as migration or mutation, are absent.

**Table 7:** Frequency of genotypes, alleles, Hardy-Weinberg balance, heterozygousity and polymorphic informative content (PIC), *GH/AluI* gene resulting from crossing several chicken lines with Arabian chickens.

Breeds	Totals	Genotype	Genotype frequency	Allele frequency	Observed/Expectation	Genotype			Allele frequency		Uji Chi- square	Heterozygosity		PIC fragment *GH* gene
		
						+ / +	+ /−	−/−	+	−		Ho	He	
SA	100	+/+	20 (0.20)	+ = 0.485, and − = 0.515	O	20	57	23	0.485	0.515	1.99^ns^	0.57	0.502	0.437
		+/−	57 (0.57)										
		−/−	23 (0.23)		E	23.52	49.96	26.52						
BA	100	+/+	26 (0.26)	+ = 0.545, and − = 0.455	O	26	57	17	0.545	0.455	2.23^ns^	0.57	0.500	0.435
		+/−	57 (0.57)										
		−/−	17 (0.17)		E	29.70	49.60	20.70						
SeA	100	+/+	24 (0.24)	+ = 0.510, and − = 0.490	O	24	54	22	0.510	0.490	0.65^ns^	0.54	0.502	0.437
		+/−	54 (0.54)										
		−/−	22 (0.22)		E	26.01	49.98	24.01						
KA	100	+/+	23 (0.23)	+ = 0.505, and − = 0.495	O	23	55	22	0.505	0.495	1.00^ns^	0.55	0.503	0.437
		+/−	55 (0.55)										
		−/−	22 (0.22)		E	25.50	50.00	24.50						
MA	100	+/+	24 (0.24)	+ = 0.500, and − = 0.500	O	24	52	24	0.500	0.500	0.16^ns^	0.52	0.503	0.437
		+/−	52 (0.52)											
		−/−	24 (0.24)		E	25,00	50,00	25,00						

SA=Super × Arab native chicken, SeA=Sentul × Arab, BA=Bangkok × Arab, KA=Kampung × Arab, MA=Merawang × Arab, *GH*=*Growth hormone*, Ho=Observed heterozygosity, He=Expected heterozygosity, ns=not significant

The PIC of the *GH*|*AluI* gene fragment (~0.437) falls within the moderate range, suggesting moder-ate genetic diversity and informativeness [45]. Higher PIC values indicate a genetically diverse population with minimal selection pressure, while lower values suggest greater homogeneity and directional selection.

Significant associations (p < 0.05) were found between GH genotypes and traits such as BW, weight gain, and back height in all crossbred groups. The (+/+) genotype was associated with superior performance across all evaluated traits (Table 8), consistent with findings from studies on GH| *BsmFI* enzyme restriction in Polish chickens [46].

**Table 8:** Association between the *growth hormone* gene and growth performance of different chicken Arabic breeds.

Crossbreed results	Traits	Genotypes		

		+/+	+/−	−/−
SA (n = 100)	Body weight (g)	1580.90 ± 182.86^a^	1410.08 ± 133.99^b^	1300.18 ± 137.82^c^
	Body weight gain (g)	485.1 ± 32.26^a^	437.2 ± 27.45^b^	380.2 ± 25.67^c^
	Back height (mm)	280.84 ± 12.58^a^	267.99 ± 8.77^b^	257.98 ± 12.04^c^
BA (n = 100)	Body weight (g)	1449.98 ± 119.09^a^	1369.26 ± 71.92^b^	1297.60 ± 106.33^c^
	Body weight gain (g)	474.70 ± 42.77^a^	427.48 ± 32.45^b^	370.11 ± 26.51^c^
	Back height (mm)	286.63 ± 26.55^a^	269.12 ± 14.89^b^	252.21 ± 25.10^c^
SeA (n = 100)	Body weight (g)	1347.42 ± 121.44^a^	1258.45 ± 94.41^b^	1108.51 ± 141.88^c^
	Body weight gain (g)	439.15 ± 38.76^a^	393.26 ± 35.76^b^	362.64 ± 24.47^c^
	Back height (mm)	264.39 ± 13.62^a^	254.48 ± 10.49^b^	241.04 ± 14.46^c^
KA (n = 100)	Body weight (g)	1333.46 ± 132.85^a^	1230.16 ± 127.65^b^	1078.47 ± 139.13^c^
	Body weight gain (g)	442.64 ± 29.27^a^	408.42 ± 24.87^b^	374.21 ± 21.56^c^
	Back height (mm)	275.49 ± 7.47^a^	263.67 ± 3.08^b^	257.57 ± 0.77^c^
MA (n = 100)	Body weight (g)	1222.39 ± 94.56^a^	1162.56 ± 68.54^b^	1056.55 ± 57.79^c^
	Body weight gain (g)	415.70 ± 39.57^a^	386.63 ± 35.57^b^	365.67 ± 18.87^c^
	Back height (mm)	225.63 ± 11.57^a^	217.20 ± 8.93^b^	201.17 ± 10.32^c^

SA=Kampung Super × Arab native chicken, BA=Bangkok × Arab, SeA=Sentul × Arab, KA=Kampung × Arab, MA=Merawang × Arab. Superscript a, b, c=Significantly different at 5% (p < 0.05) for each genotype in chicken breeds; The numbers shown in parentheses are the number of individuals with the specified breed

This study is among the first in Indonesia to combine large-scale phenotyping and *GH* gene polymorphism analysis in Arabic crossbred chickens. The results provide a foundation for future MAS. As noted by List *et al*. [47], GH is involved in a wide array of physiological processes, including postnatal growth, protein synthesis, adiposity, and reproductive performance.

## CONCLUSION

This study comprehensively evaluated the growth performance, egg traits, morphometric characteristics, and *GH* gene polymorphism in crossbred chickens derived from local Indonesian breeds and Arabian chi-ckens. The SA cross consistently outperformed other crossbreds (BA, SeA, KA, and MA) across multiple metrics, including BW, weight gain, morphometric dimensions, and egg characteristics. Notably, BW gain peaked between 2 and 3 months of age across all groups, highlighting an optimal growth window for intervention. PCA identified back height as the domi- nant morphometric trait influencing body confor-mation, and the homozygous GH genotype (+/+) was significantly associated with improved growth performance and morphometric outcomes.

The findings offer valuable insights for poultry breeders aiming to enhance productivity in native chicken populations. The integration of phenotypic selection with GH genotyping can enable earlier and more accurate identification of superior birds, improving efficiency in breeding programs. The moderate poly-morphism of the GH|AluI marker also demonstrates its potential utility for MAS in resource-limited settings.

This study is among the first in Indonesia to combine large-scale morphometric, performance, and genotypic analyses in indigenous × Arabian crossbred chickens under controlled environmental conditions. The use of robust statistical models and controlled rear-ing enhances the reliability of genotype–phenotype associations.

The study was limited to the first generation (G1) of crossbreds and focused only on a single growth-related gene (*GH*). Environmental interactions were standardized, which may not fully reflect field variability across different production systems.

Further investigations should evaluate multigen-erational effects of *GH* gene polymorphism and explore additional candidate genes linked to growth and reproductive performance. Incorporating transcriptomic and proteomic tools may elucidate underlying mole-cular mechanisms. Field validation of MAS in varied agroecological zones is also recommended to ensure scalability.

The integration of phenotypic traits and GH genotyping in crossbred chickens provides a scientific foundation for genetic improvement strategies in tropi-cal poultry production. This research strengthens the case for implementing MAS to sustainably enhance the productivity and genetic potential of indigenous chicken populations.

## DATA AVAILABILITY

All the generated data are included in the manuscript.

## AUTHORS’ CONTRIBUTIONS

DD, GG, and RSH: Study conception and design and data acquisition. DD and RSH: Conducted fieldwork and laboratory investigations. RAM, AA, YA, and SEW: Literature review, data analysis, and interpretation. DD, RSH, and SEW: Drafted the manuscript and prepared the figures and tables. GG, RAM, AA, and YA: Revised the manuscript. All authors have read and approved the final manuscript.

## References

[1] Pagala M.A, Saili T, Nafiu L.O, Sandiah N, Baa L.O, Aku A.S, Zulkarnaen D, Kurniawan W (2017). Polymorphism of Mx|Hpy81 genes in native chickens observed using the PCR-RFLP technique. Int. J. Poult. Sci.

[2] Khaerunnisa I, Pramujo M, Arief II, Budiman C, Gunawan A, Jakaria J, Sumantri C (2016). Polymorphism of the T4842G myostatin gene is associated with carcass characteristics in Indonesian chickens. Int. J. Poult. Sci.

[3] Alwi W, Agustina L, Mide M.Z (2019). Arabic chicken (Gallus turcicus) performance with different dietary energy-protein levels. [Performa Ayam Arab dengan pemberian energi-protein pada level berbeda.*J. Sains. Teknol. Peternakan*]. JSTP.

[4] Putri A.B.S.R, Depison G (2020). Body weight and morphometric characteristics of several local chicken lines [Bobot badan dan karakteristik morfometrik beberapa galur ayam local]. J. Trop. Anim. Sci. Technol.

[5] Abdu M, Gushairiyanto G, Depison D (2021). The relationship of egg weight with DOC weight and DOC weight with body weight of first generation of Sentul chicken (G1). J. Ilmi. Peternakan Terpadu.

[6] Rahayu F.F, Depison D, Gushairiyanto G (2021). Performance of Kampung super chicken and Bangkok chicken first generation (G1) until the age of 12 weeks. Livest. Anim. Res.

[7] Depison W, Gushariyanto G (2022). Comparison of productivity of sentul and kampung chickens until the age of 3 months in the first-generation selection population (G1). Bul. Peternakan.

[8] Irmaya D, Depison D, Gushairiyanto G (2021). Quantitative characteristic of Indonesian native chickens at the age of 4 months. Livest. Anim. Res.

[9] Prawira R, Depison D, Gushariyanto G, Erina S (2021). The relationship between egg morphology with egg weight and DOC weight with body weight of Kampung F1 [Hubungan morfologi telur dengan bobot telur dan bobot DOC dengan bobot badan ayam kampung F1]. J. Ilmu Peternakan Terapan.

[10] Sari M, Depison D, Gushariyanto G, Wiyanto E (2021). Relationship between egg weight and hatching weight and hatching weight and body weight of G1 Merawang chickens up to 4 months of age [Hubungan bobot telur dengan bobot tetas dan bobot tetas dengan bobot badan ayam merawang G1 sampai umur 4 bulan]. J. Peternakan.

[11] Gultom L.H.M, Gushairiyanto G, Depison D (2021). Correlation of sentul chicken body weight at DOC age of 1, 2 and 3 months. J. Sain Peternakan Indonesia.

[12] Assefa H, Melesse A (2018). Morphological and morphometric characterization of indigenous chicken populations in the Sheka Zone, Southwestern Ethiopia. Poult. Fish. Wildl. Sci.

[13] Putranto H.D, Setianto J, Yumiati Y, Handika D (2018). Analyses of body and chest morphometric comparison between two Indonesian local poultry species. Int. J. Agricul. Technol.

[14] Schreiter R, Freick M (2023). Laying performance characteristics, egg quality, and integument condition of Saxonian chickens and German Langshan bantams in a free-range system. J. Appl. Poult. Res.

[15] Kazemi H, Rezaei M, Hafezian H, Rahimi Mianji G, Najafi M (2018). Genetic analysis of SNPs in GH, GHR, IGF-I and IGFBPII genes and their association with some productive and reproductive traits in native breeder hens. Gene Technol.

[16] Anh N.T.L, Kunhareang S, Duangjinda M (2015). Association of chicken growth hormones and insulin-like growth factor gene polymorphisms with growth performance and carcass traits in Thai broilers. Asian-Australas J. Anim. Sci.

[17] Kadlec J, Hosnedlová B, Řehout V, Čítek J, Večerek L, Hanusová L (2011). Insulin-like growth factor-I gene polymorphism and its association with growth and slaughter characteristics in broiler chickens. J. Agrobiol.

[18] Hosnedlova B, Vernerova K, Kizek R, Bozzi R, Kadlec J, Curn V, Kouba F, Fernandez C, Machander V, Horna H (2020). Associations between IGF1, IGFBP2 and TGFß3 genes polymorphisms and growth performance of broiler chicken lines. Animals (Basel).

[19] Nei M, Kumar S (2000). Molecular Evolution and Phylogenetics.

[20] Hartl D.L, Clark G.C (1997). Principles of Popula-tion Genetics.

[21] Sola-Ojo F.E, Ibiwoye D.I, Akilapa M.A (2020). Effects of strain on body weight and morphometric traits and their relationships in four broiler chicken types during the starter and finisher stages. J. Agricul. Food Environ.

[22] Chomchuen K, Tuntiyasawasdikul V, Chankitisakul V, Boonkum W (2022). Genetic evaluation of body weights and egg production traits using a multi-trait animal model and selection index in Thai native synthetic chickens (kaimook e-san2). Animals (Basel).

[23] Saili T, Nafiu L.O, Bain A, Asminaya N.S, Badaruddin R, Lestari Y (2024). Quality characteristics of eggs-derived superior native chicken in Kendari city. In:Technological Innovations in Tropical Livestock Development for Environmental Sustainability and Food Security.

[24] Pagala M.A, Indi A, Badaruddin R, Sandiah N, Aprianti N (2020). The egg fertility from offspring of crossbreeding results of Bangkok chickens and laying hens. IOP Conf. Ser. Earth Environ. Sci.

[25] Ishak L.O.A.K, Saili T, Rusdin M (2023). Characteristics of kampong chicken (*Gallus domesticus*) eggs based on shank color. Indones. J. Anim. Agric. Sci.

[26] Sutiyo R.A, Wirapartha M, Dewi G.A.M.K (2020). The effect of salt spraying on the hatchability performance of the hybrid duck eggs. J. Peternakan Tropika.

[27] Sunarno S, Budiraharjo K, Solikhin S (2021). Analysis of the effects of intensive and extensive maintenance systems on the productivity and quality of Tegal duck eggs [Analisis efek pemeliharaan sistem intensif dan ekstensif terhadap produktivitas dan kualitas telur itik tegal]. J. Peternakan Indones. (Indones. J. Anim. Sci).

[28] Zhang J, Gao X, Zheng W, Wang P, Duan Z, Xu G (2023). Dynamic changes in egg quality, heritability and correlation of these traits and yolk nutrient throughout the entire laying cycle. Foods.

[29] Noor R.R (2010). Genetika Ternak.

[30] Rowiyanti W.O, Junaedi J, Suparman S (2022). Bodyweight growth of chicken from cross breed of Kampung chicken with Bangkok chicken [Pertumbuhan bobot badan ayam hasil persilangan ayam Kampung dengan ayam Bangkok]. J. Sains Teknol. Peternakan.

[31] Subiharta S, Prabowo A (2020). Evaluation of the grade of hatching eggs of Sensi-1 Agrinak and KUB crossbred chicken on hatching and production performances [Pengkajian penggemukan ayam persilangan ayam sentul terseleksi-KUB (SENKUB) dalam mendukung kecukupan pangan asal ternak]. In:Prosiding Seminar Nasional Kesiapan Sumber Daya Pertanian dan Inovasi Spesifik Lokasi Memasuki Era Industri 40. Balai Besar Pengkajian dan Pengembangan Teknologi Pertanian, Bogor,.

[32] Garel M.E.C, Philippe V (2022). Estimating of additive, dominance, and epistatic genetic variance in eucalypt hybrid population. Silvae Genet.

[33] Nguyen H.T.H, Chen Z.Q, Fries A, Berlin M, Hallingbäck H.R, Wu H.X (2022). Effect of additive, dominant and epistatic variances on breeding and deployment strategy in Norway spruce. For. Int J. For. Res.

[34] Mancinelli A.C, Menchetti L, Birolo M, Bittante G, Chiattelli D, Castellini C (2023). Crossbreeding to improve local chicken breeds:Predicting growth performance of the crosses using the Gompertz model and estimated heterosis. Poult. Sci.

[35] Hailemariam A, Esatu W, Abegaz S, Urge M, Assefa G, Dessie T (2023). Effects of crossbreeding on growth, production and selected egg quality traits of improved Horro crosses with cosmopolitan chickens. J. Agric. Food Res.

[36] Jacob F, Salgado D, Nää I, Baracho M (2020). Effect of environmental enrichment on the body weight in broiler chickens. Braz. J. Poult. Sci.

[37] Kholik A, Indrijani H, Tanwiriah W (2019). Modelling growth curves in Kampung super garut chicken fed with pasak bumi meal. (Euricoma longifolia Jack).*J. Anim. Sci. Padjadjaran Univ*.

[38] Utama M, Depison Gushairiyanto, Ediyanto H (2022). Comparison of fertility, hatchability and quantitative characteristics between KUB chicken and Kampung chicken (G1) [Perbandingan daya tunas, daya tetas, dan karakteristik kuantitatif ayam KUB dengan ayam kampung (G1)]. J. Trop. Anim. Sci. Technol.

[39] Mariandayani H.N, Darwati S, Khaerunnisa I, Prasasty V.D (2023). Growth performance of Indonesian three-breed cross chicken associated with growth hormone and insulin-like growth factor 2 genes. Vet. World.

[40] Güzel B.C, Manuta N, Ünal B, Ruzhanova-Gospodinova I.S, Duro S, Gündemir O (2024). Size and shape of the neurocranium of laying chicken breeds. Poult. Sci.

[41] Milas E.S.S, Saerang J.L.P, Lambey L.J, Takaendengan B.J (2020). Phenotypic characteristics of some quantitative traits of native chickens in Minahasa [Karakteristik fenotipe beberapa sifat kuantitatif ayam kampung di Minahasa]. ZOOTEC.

[42] Mustefa A, Kenfo H, Belayhun T, Hailu A, Assefa A (2021). Morphometric and morphological characterization of chicken resources adapted to pastoral and agropastoral areas of Southern Ethiopia. Gen. Resour.

[43] Allendorf F.W, Luikart G, Aitken S.N (2013). Conservation and the Genetics of Populations.

[44] Klug H (2024). Mating systems, a brief history of. In:Reference Module in Life Sciences. Elsevier, Netherlands.

[45] Serrote C.M.L, Reiniger L.R.S, Silva K.B, Rabaiolli S.M.D.S, Stefanel CM (2020). Determining the polymorphism information content of a molecular marker. Gene.

[46] Mazurowski A, Frieske A, Kokoszyński D, Mroczkowski S, Bernacki Z, Wilkanowska A (2015). Examination of growth hormone (*GH*) gene polymorphism and its association with body weight and selected body dimensions in ducks.. Folia Biol. (Praha).

[47] List E.O, Berryman D.E, Jensen E.A, Kulkarni P, McKenna S, Kopchick J.J (2020). New insights of growth hormone (GH) actions from tissue-specific GH receptor knockouts in mice. Arch. Endocrinol. Metab.

